# Molecular Characterization of Thyroid Toxicity: Anchoring Gene Expression Profiles to Biochemical and Pathologic End Points

**DOI:** 10.1289/ehp.7690

**Published:** 2005-05-12

**Authors:** Christine M. Glatt, Ming Ouyang, William Welsh, John W. Green, John O Connor, Steven R. Frame, Nancy E. Everds, Greg Poindexter, Suzanne Snajdr, Don A. Delker

**Affiliations:** 1 DuPont Haskell Laboratory, Newark, Delaware, USA; 2 Department of Pharmacology, Robert Wood Johnson Medical School and Informatics Institute of the University of Medicine and Dentistry of New Jersey, Piscataway, New Jersey, USA

**Keywords:** excess iodide, gene expression, microarrays, oxidative stress, phenobarbital, propylthiouracil, thyroid, Wnt signaling

## Abstract

Organic iodides have been shown to induce thyroid hypertrophy and increase alterations in colloid in rats, although the mechanism involved in this toxicity is unclear. To evaluate the effect that free iodide has on thyroid toxicity, we exposed rats for 2 weeks by daily gavage to sodium iodide (NaI). To compare the effects of compounds with alternative mechanisms (increased thyroid hormone metabolism and decreased thyroid hormone synthesis, respectively), we also examined phenobarbital (PB) and propylthiouracil (PTU) as model thyroid toxicants. Follicular cell hypertrophy and pale-staining colloid were present in thyroid glands from PB-treated rats, and more severe hypertrophy/colloid changes along with diffuse hyperplasia were present in thyroid glands from PTU-treated rats. In PB-and PTU-treated rats, thyroid-stimulating hormone (TSH) levels were significantly elevated, and both thyroxine and triiodothyronine hormone levels were significantly decreased. PB induced hepatic uridine diphosphate-glucuronyltransferase (UDPGT) activity almost 2-fold, whereas PTU reduced hepatic 5′-deiodinase I (5′-DI) activity to < 10% of control in support of previous reports regarding the mechanism of action of each chemical. NaI also significantly altered liver weights and UDPGT activity but did not affect thyroid hormone levels or thyroid pathology. Thyroid gene expression analyses using Affymetrix U34A GeneChips, a regularized *t*-test, and Gene Map Annotator and Pathway Profiler demonstrated significant changes in rhodopsin-like G-protein–coupled receptor transcripts from all chemicals tested. NaI demonstrated dose-dependent changes in multiple oxidative stress–related genes, as also determined by principal component and linear regression analyses. Differential transcript profiles, possibly relevant to rodent follicular cell tumor outcomes, were observed in rats exposed to PB and PTU, including genes involved in Wnt signaling and ribosomal protein expression.

Thyroid cancer, a fairly uncommon form of cancer in the human population, has causal links to environmental radiation exposures (Meirmanov et al. 2003). Although there are multiple epidemiology studies that associate radiation exposure with thyroid cancer, there have been no studies to associate human thyroid cancer with environmental chemical exposures (Hard 1998). The human thyroid responds toxicologically to multiple antithyroid drugs, including excess iodide, propylthiouracil (PTU), and thionamides, but the progression to cancer after repetitive administration has not been observed (Hard 1998; Hill et al. 1998; Markou et al. 2001). In contrast, multiple antithyroid drugs administered to the rat have demonstrated an increase in thyroid tumors, including PTU, thionamides, and the hepatic enzyme inducer phenobarbital (PB) (Capen 1997; Hurley et al. 1998; McClain et al. 1988). Other xenobiotics, including several organic iodides such as amiodarone and erythrosine, have also been associated with thyroid tumor development in the rat. Many organic iodides alter rat thyroid homeostasis, causing thyroid hypertrophy and alterations in colloid that may potentially lead to thyroid tumors after chronic administration (Capen 1997; Hurley et al. 1998).

The mechanisms by which organic iodides induce thyroid toxicity are varied and may include excess iodide being released into the blood during xenobiotic metabolism, toxicity to the liver that alters thyroid hormone metabolism, and/or direct thyroid toxicity that inhibits the release of thyroid hormones into the circulation (Capen 1997; Hurley et al. 1998). In subchronic toxicology studies it has been difficult to predict whether early changes in thyroid pathology could lead to thyroid cancer in the rat after chronic administration of organic iodides. Excess iodide alone can be toxic to thyroid cells in culture and cause thyroid hypertrophy and changes in colloid *in vivo* in the rat model (Capen 1997; Vitale et al. 2000). It has also been reported that administration of excess iodide promotes thyroid tumor development in rats initiated with *N*-bis(2-hydroxypropyl)-nitrosamine (Kanno et al. 1992). However, iodide excess alone has not been sufficient in inducing follicular cell hyperplasia and thyroid tumors in rats but more commonly causes hypothyroidism (Backer and Hollowell 2000; Kanno et al. 1994). This is believed to be due partly to the escape from the Wolff-Chaikoff effect (acute inhibition of iodine organification) that is seen within days of exposure to excess iodide (Wolff and Chaikoff 1948). This escape phenomenon is associated with the down-regulation of the sodium iodide (NaI) symporter (NIS) thereby reducing the amount of inorganic iodine in the thyroid so that thyroxine (T_4_) and thyroid-stimulating hormone (TSH) secretions are returned to normal physiological levels (Eng et al. 1999).

Chronic elevation of TSH levels has been associated with an increased risk of thyroid tumors in the rat, which may be due, in part, to the high turnover of the circulating thyroid hormone triiodothyronine (T_3_) in this species compared with the lower T_3_ turnover rate in humans (Capen 1997). Many antithyroid drugs and PB mediate their carcinogenic properties by elevating circulating TSH levels in the rat albeit by different mechanisms (Hood et al. 1999; McClain et al. 1988). Although PTU reduces thyroid hormone production by inhibiting thyroglobulin organification in the thyroid and inhibiting the peripheral conversion of T_4_ to active T_3_, PB reduces circulating T_4_ by increasing its hepatic metabolism and excretion via glucoronidation. Reductions in thyroid hormone (T_4_ and T_3_) by either mechanism causes an elevation in TSH that is sufficient in causing thyroid tumors in rats after prolonged exposure without any evidence of thyroid DNA damage (McClain 1992). Organic iodides may also increase TSH levels by similar mechanisms; however, it is unknown what contribution iodide excess has in their overall toxicity to the rat thyroid. To this end we have implemented biochemical, pathological, and molecular analyses to characterize the rat thyroid response to three model toxicants: excess iodide by using NaI a noncarcinogen, and the rodent thyroid carcinogens PB and PTU, in a modified 2-week endocrine battery (O’Connor et al. 2002). The goals of this study were to identify dose-dependent gene expression profiles induced by excess iodide in rats, determine whether gene expression profiles could be obtained that correlate with clinical and pathological end points in rats, and determine whether profiles are predictive of the carcinogenic potential of each chemical in rats.

## Materials and Methods

### *In Vivo* Studies

Adult male Crl:CD (SD)IGS BR rats, approximately 8 weeks of age, were treated with NaI, PB, and PTU for 14 consecutive days. NaI, PB, and PTU were purchased from Sigma Chemical Company (St. Louis, MO). Rats (*n* = 20/group) were dosed by oral gavage with vehicle (water or 0.25% methylcellulose), NaI (0.1, 1, 10, or 100 mg/kg/day), PB (100 mg/kg/day), or PTU (10 mg/kg/day) at a dose volume of 5 mL/kg. NaI was dissolved in water, whereas PB and PTU were dissolved in methylcellulose. On day 15, all rats were euthanized by carbon dioxide anesthesia and exsanguination. Blood samples were collected from the inferior vena cava of each animal at necropsy to measure serum levels of TSH, T_4_, T_3_, and reverse T_3_ (rT_3_). Terminal body, thyroid gland, and liver weights were recorded for the first 10 animals of each dose group. The thyroid gland and surrounding tissue from the first 10 animals of each dose group were processed for histopathological evaluation. A liver sample from the first five animals of each dose group was processed to measure 5′-deiodinase I (5′-DI) and uridine diphosphate-glucuronyltransferase (UDPGT) activity. Thyroid glands from the last 10 animals (five from methylcellulose group) from each dose group were removed and placed in RNALater (Ambion, Austin, TX) overnight at 4°C. The next day, thyroids were removed from the RNALater and stored at –80°C until processed for total RNA.

The research described in this publication was conducted in a laboratory accredited by the Association for the Assessment and Accreditation of Laboratory Animal Care International, and the investigators complied with the regulations and standards of the Animal Welfare Act and adhered to the principles of the *Guide for the Care and Use of Laboratory Animals* (National Research Council 1996).

### Pathological Evaluations

After euthanization the thyroid glands and surrounding tissue from the first 10 animals from each group were removed and placed into formalin fixative for at least 48 hr before trimming and weighing. After fixation, one individual performed a final dissection under a dissecting microscope. This was done in order to reduce the variability of the dissection procedure, thereby reducing the variability of the thyroid gland weights. Organ weights were calculated relative to body weight. The formalin-fixed thyroid glands were examined microscopically.

### Hormonal Measurements

Blood was collected at the time of euthanization from all animals. Serum was prepared and stored between –65°C and –85°C until analyzed for serum hormone concentrations. Serum TSH (Amersham Biosciences Corp., Piscataway, NJ), T_3_ and T_4_ (Diagnostic Products Corp., Los Angeles, CA), and rT_3_ (Polymedco Corp., Cortlandt Manor, NY) concentrations were measured using commercially available RIA kits.

### Microsomal Preparations

At necropsy, a section of the liver from the first five animals from each group was removed, and hepatic microsomes were prepared for biochemical evaluation. A portion of the liver was homogenized (1 g tissue/8 mL buffer) in buffer containing 50 mM Tris–HCl, 0.25 M sucrose, and 5.4 mM EDTA, pH 7.4. The homogenates were centrifuged at 15,000 × *g* for 15 min at 4°C. The resulting supernatants were removed and centrifuged at 100,000 × *g* for 70 min at 4°C; these pellets contained the microsomal fractions. The microsomal pellets were resuspended in the homogenization buffer at a protein concentration of 10–20 mg/mL, aliquoted, and stored between –65°C and –85°C until analyzed for UDPGT and 5′-DI. The protein content of the microsomes was measured before and after analyses by the BioRad method (Bradford 1976). Final calculations were based on the postassay protein determination.

### 5′-Deiodenase I Measurements

Microsomal 5′-DI activity was determined using modifications of the methods of Pazos-Moura et al. (1991) and Leonard and Rosenberg (1980). Briefly, 50 μL of reaction mixture [0.1 M potassium phosphate, 1 mM EDTA, 0.5 mM DTT, 14.5 μL [^125^I]rT_3_ (~ 4.9 μCi, 0.05 nmol)] was preincubated at 37°C for approximately 1 min. Fifty micro-liters of microsomes, diluted with liver homogenization buffer to achieve a final protein concentration of 0.5 mg/mL, was added to the reaction mixture. The tubes were vortexed and incubated for 10 min at 37°C. The reaction was stopped by the addition of 33 μL of ice-cold BSA–PTU solution (4% BSA, 5 mM PTU) and 133 μL of 20% TCA, and the tubes were placed on ice until centrifugation. All tubes were centrifuged for 3 min at 12,000 × *g*, 4°C. Two hundred microliters of the resulting supernatant (75% of the reaction volume) was applied to a Poly-Prep column equilibrated with 10% acetic acid, and eluted with 1.7 mL of 10% acetic acid. The resulting eluate was measured on a gamma counter to determine 5-DI activity (nmoles [^125^I]rT_3_ deiodinated per hour per milligram protein).

### UDPGT Measurements

Microsomal UDPGT activity was determined spectrophotometrically using a modification of the method of Bock et al. (1983). Briefly, 15 μL of *p*-nitrophenol (66.7 mM) was added to 460 μL of microsomes, which had been resuspended with assay buffer [66 mM Tris–HCl, 10 mM magnesium chloride, 0.05% Brij 58 (polyoxyethylene ether 2 cetyl ether; CAS no. 9004-95-9), pH 7.5] to achieve a final protein concentration of 0.5–1.0 mg/mL. The tubes were preincubated at 37°C for 2 min before the addition of 25 μL UDPGA (uridine diphosphate glucuronic acid; 200 mM) to start the reaction. The tubes were vortexed and incubated for 10 min at 37°C. A reaction blank tube for each sample was run concurrently by substituting the UDPGA with assay buffer. The reaction was stopped by the addition of 0.5 mL ice-cold methanol, and the tubes were placed on ice until centrifugation. All tubes were centrifuged for 10 min at 2,000× *g* at 4°C. Three hundred microliters of the supernatant was combined with 2.7 mL 0.1 N sodium hydroxide, and the absorbance at 405 nm was measured. Rate of UDPGT activity is expressed as nmoles per minute per milligram protein.

### Microarray Analysis

RNA preparation and analysis was done according to the Affymetrix-recommended protocol (Affymetrix 2002). Briefly, total RNA from four animals from each dose group was prepared individually using the TRIzol procedure (Invitrogen, Carlsbad, CA) and cleaned using the Qiagen RNeasy mini RNA cleanup protocol (Qiagen, Valencia, CA). The integrity of each RNA sample was determined using an Agilent 2100 Bioanalyzer (Agilent, Foster City, CA). After this, double-stranded cDNA from three of the four samples was prepared from 16 μg of total RNA using Superscript II reverse transcriptase (Invitrogen) and a T7 primer (Genset, Boulder, CO) for first-strand synthesis, and DNA polymerase and ligase (Invitrogen) for second-strand synthesis. Subsequently, labeled cRNA was synthesized from the cDNA using the Enzo RNA transcript labeling kit (Affymetrix, Santa Clara, CA) according to the manufacturer’s instructions. Approximately 20 μg of biotin-labeled cRNA was then fragmented in a solution of 40 mM Tris–acetate, pH 8.1, 100 mM KOAc, and 30 mM MgOAc at 94°C for 35 min. Labeled cRNA was hybridized to the Affymetrix GeneChip Test2 Array (Affymetrix) to verify the quality of labeled cRNA. After this, cRNA was hybridized to the Affymetrix Rat Genome U34A GeneChip Probe Array (RG-U34A; Affymetrix). The cRNA in hybridization cocktail was incubated overnight at 45°C while rotating in a hybridization oven. After approximately 16 hr of hybridization, the cocktail was removed and the arrays were washed and stained in a Fluidics Station 400 (Affymetrix) according to the Affymetrix-recommended protocol (Affymetrix 2002). Briefly, several cycles of washes were done initially with a nonstringent buffer (1 M NaCl, 67 mM NaH_2_PO_4_, 6.7 mM EDTA, 0.01% Tween 20) at 25°C and then with stringent buffer [100 mM MES, 0.1 M Na^+^, 0.01% Tween 20] at 50°C. The arrays were then stained in streptavidin phycoerythrin (SAPE) solution (10 μg/mL SAPE, 2 mg/mL acetylated BSA, 100 mM MES, 1 M Na^+^, 0.05% Tween 20) at 25°C, washed in nonstringent buffer, stained in antibody solution (2 mg/mL acetylated BSA, 100 mM MES, 1 M [Na^+^], 0.05% Tween 20, 0.1 mg/mL normal goat IgG, 3 μg/mL anti-streptavidin biotinylated antibody) at 25°C, stained again in SAPE solution at 25°C, and then washed again in nonstringent buffer at 30°C. Arrays were then scanned on a GeneArray scanner (Agilent). Image analysis, quantification of raw gene expression values, mismatched probe background subtraction, and present/absent calls were performed using the Microarray Suite software (version 5.0; Affymetrix).

### Data Analysis

Differential gene expression was determined by the regularized *t*-test, which uses a Bayesian procedure (Baldi and Long 2001). Briefly, the expression level of each gene is assumed to be from a normal distribution with μ and σ^2^. Using a conjugate prior, the mean of the posterior (MP) estimate of μ is the sample mean. The MP estimate of σ^2^ is


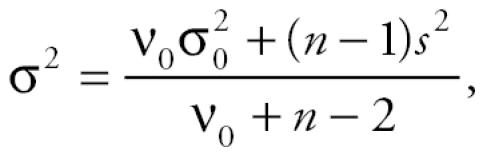


where *n* is the sample size, *s* ^2^ is the sample variance, *v*_0_ is the degrees of freedom of the prior (a value of 10 is used in the analysis), and σ _0_^2^ is the mean of sample variances of genes in the neighborhood of the gene under consideration. The neighborhood is the 50 genes with sample means immediately above and below the sample mean of the gene under consideration; that is, the neighborhood consists of the 101 genes centered on the gene. After the MP estimates of μ and σ^2^ are obtained, the *t*-test of unequal variances is used to calculate a *p*-value of differential expression.

Multiple linear regressions are used to determine dose-dependent expression after NaI treatments of 0.1, 1, 10, or 100 mg/kg/day. Some genes respond to NaI linearly, but for other genes, the induction or repression of expression may become saturated after some dose levels. Therefore, two types of multiple linear regressions were performed. The first type was the linear regression of the gene expression levels and the dose levels, and the other type was the linear regression of the gene expression levels and the logarithms of the dose levels.

A principal component analysis was also performed on the data. Three animals were measured within each treatment for each gene. The treatment means were then subjected to principal component analysis. The components were thus determined on a per-treatment basis rather than a per-gene basis, as in Raychaudhuri et al. (2000). For each of the treatments, the mean of the transcription signals was computed. Then, for each treatment, the ratio of the treatment mean to the corresponding control mean was computed. The logarithms of these six signal ratios were then analyzed. There was no need to standardize these log-ratios, because they were essentially standardized without further attention.

## Results

### Liver Weights and Hormone Metabolism

**Figure 1:**
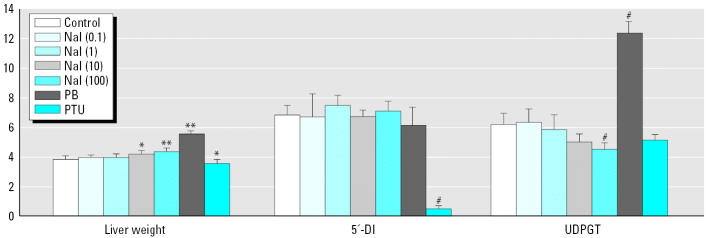
Hepatic metabolism parameters: mean ± SD of 5–10 measurements from individual animals. Liver weight is expressed as percent body weight. 5′-DI activity is expressed as nmol [^125^I]rT_3_ deiodinated/hr/mg protein. UDPGT activity is expressed as nmol/min/mg protein/10. *Significant by least significant difference. **Significant by least significant difference and Dunnett’s test. ^#^Significant by Dunnett/Tamhane-Dunnett test.

After the 2-week exposure period, liver weights were increased in a dose-dependent manner and were significantly higher in rats administered 10 and 100 mg/kg/day NaI (8–13% increase) and 100 mg/kg/day PB (44% increase) compared with control rats that received water alone (Figure 1). Liver weights were slightly reduced in animals that received 10 mg/kg/day PTU, and this was attributed to concomitant reductions in body weight in this treatment group (data not shown).

UDPGT activity was significantly higher (99% increase) in rats administered 100 mg/kg/day PB compared with controls (Figure 1). In contrast, UDPGT activity was reduced (28% decrease) in rats that received 100 mg/kg/day NaI for 2 weeks. 5′-DI activity was dramatically reduced in PTU-treated animals (93% decrease), whereas all other treatments had no significant change in activity compared with controls (Figure 1).

### Thyroid Hormone Levels and Histopathology

Treatment-related effects on thyroid hormone levels were observed in the 100 mg/kg/day PB and 10 mg/kg/day PTU groups. Compared with controls, T_3_, T_4_, and rT_3_ levels were reduced 23, 40, and 28%, respectively, in PB-treated rats and 80, 99, and 56%, respectively, in PTU-treated rats (Table 1). TSH levels were increased approximately 2- and 4-fold in PB- and PTU-treated rats, respectively. There were no treatment-related effects on thyroid hormone levels observed with NaI at any concentration tested.

Treatment-related changes in thyroid gland histopathology were observed in the PB and PTU treatment groups (Table 2). Thyroid follicular cell hypertrophy and pale-staining colloid were observed in both PB and PTU treatment groups, and diffuse hyperplasia was observed in the PTU group. Relative thyroid gland weights (percent of body weight) were also significantly increased (~ 3-fold) in the PTU treatment group compared with controls (Table 2). No treatment-related changes in thyroid gland histopathology were observed after NaI administration at any of the doses tested.

### Thyroid Gland Gene Expression

#### Principal component analysis.

Thyroid gene expression data were analyzed using principal component analysis, a regularized t-test and multiple linear regressions. Principal component analysis of gene expression data from all 24 samples demonstrated grouping according to treatment. Six principal components were identified (Table 3). Of these, the first four account for approximately 85% of the total variation in the data. Neither of the remaining two meets the 70/N rule used by Raychaudhuri et al. (2000). The general methodology of that reference was followed in the present analysis. To understand these principal components, it is helpful to express each as a linear combination of the means of the six treatments (Table 4). The first component is essentially the average of the NaI treatment log-signal ratios. Large values of this component tend to be associated with up-regulation in one or more NaI treatments. Large negative values (i.e., negative numbers large in absolute value) of this component tend to be associated with down-regulation in one of more NaI treatments. The second component is an indicator for an effect due to PB and PTU. A large value of Pcomp2 (principal component 2) indicates an up-regulation, whereas a large negative value indicates a down-regulation. Component 3 is primarily a contrast between the PB and PTU treatments. The fourth principal component is primarily an indicator of effect at low doses of NaI. Based on these principal component analysis findings, further gene ontology work was directed to the first two principal components, namely, genomic profiles associated with NaI exposure or PB and PTU exposure.

#### Multiple linear regressions.

**Figure 2:**
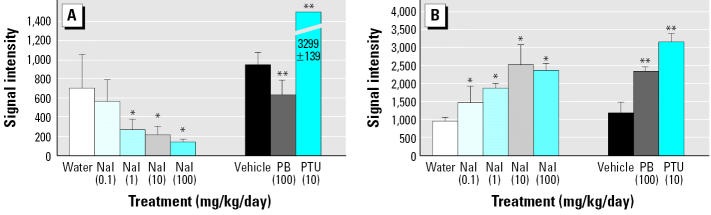
Bar graph of dose-dependent NIS (A) and GST-pi (B) gene expression. Values represent mean ± SD of three measurements from individual animals. Significantly different at *p < 0.05 from water and **p < 0.05 from vehicle controls by regularized t-test.

Dose-dependent expression, as determined by multiple linear regressions, was observed after NaI treatment. Transcript levels most influenced by dose (p ≤0.001), included the NIS [Slc5a5; GenBank accession no. U60282; (http://www.ncbi.nlm.gov)] and antioxidant enzymes such as glutathione peroxidase 2 (Gpx2), thioredoxin reductase (Txnrd1), and glutathione S-transferase pi (GST-pi; Gstp2) (Table 5). NIS mRNA was down-regulated in a dose-dependent manner to 80 (not statistically significant), 40, 30, and 20% of control values after 0.1, 1, 10, and 100 mg/kg/day NaI, respectively (Figure 2A). However, unlike NaI, PTU increased NIS expression by greater than 300% of control, and PB only slightly reduced (70% of control) NIS transcript levels. The most statistically significant of the antioxidant enzymes, GST-pi, was up-regulated to 154, 196, 263, and 250% of control values after the same NaI dosing regimen (Figure 2B). Similar to NaI, PB and PTU increased GST-pi expression levels to 198 and 265% of control, respectively.

#### Regularized t-test (Bayesian procedure).

In a separate analysis using the regularized t-test, 872, 948, and 1552 gene transcripts (of 8,740 transcripts present in all samples) were significantly (p < 0.01) changed by 100 mg/kg/day NaI, 100 mg/kg/day PB, and 10 mg/kg/day PTU administration compared with controls, respectively. To further characterize these genomic changes according to biological function and identify molecular pathways involved in the mode of action of each chemical, these gene lists were uploaded into GenMAPP (Gene Map Annotator and Pathway Profiler, version 1.0; Dahlquist et al. 2002). Two molecular pathways found to be highly influenced by NaI were rhodopsin-like G-protein receptors and oxidative-stress–related genes (Table 5). These pathways were also influenced by PB and PTU treatment, although the spectrum of gene transcripts was unique for each chemical. Although all chemicals affected the expression of one or more adrenergic receptors, dopamine and chemokine receptor transcripts were only affected by PB and PTU treatment, respectively. PB and PTU also caused a down-regulation of TSH receptor mRNA (Table 5) correlating with the increased circulating TSH observed with these chemicals from the serum hormone analysis. Interestingly, NaI induced specific changes in multiple olfactory receptor and oxidative stress transcripts, including Txnrd1, Gpx2, superoxide dismutase 2 (Sod2), and nitric oxide synthase 2 (Nos2), that were not affected by PB or PTU treatment (Table 5). NaI also altered the levels of multiple transcripts involved in apoptosis that were not affected by PB or PTU treatment including receptor-interacting serine-threonine kinase 3 [Ripk3, GenBank accession no. AF036537 (http://www.ncbi.nlm.nih.gov)], BH3 interacting domain 3 [Bid3; GenBank accession no. AI102299 (http://www.ncbi.nlm.nih.gov)], and scavenger receptor class B [Scarb1; GenBank accession no. D89655 (http://www.ncbi.nlm.nih.gov); p < 0.001]

**Figure 3:**
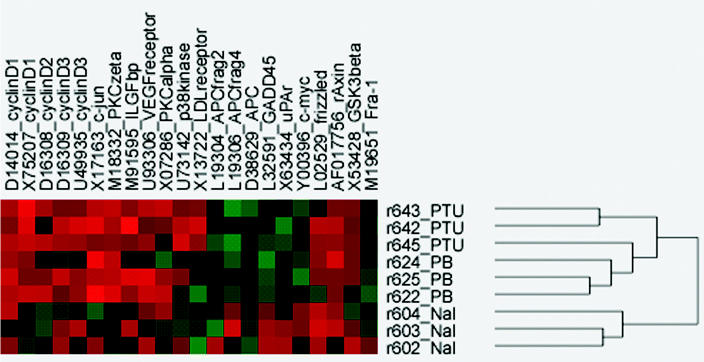
Agglomerative clustering of treated animals (r602–r604, NaI 100 mg/kg; r622–r625, PB 100 mg/kg; r642–r645, PTU 10 mg/kg; n = 3/group) based on individual expression values of Wnt signaling genes. Values represent the log_2_ of the fold change (each animal value divided by the control mean). Log_2_ values were uploaded into EPCLUST [Expression Profiler, European Bioinformatics Institute (http://www.ebi.ac.uk/expressionprofiler)] and clustered by Euclidean distance. Numerical values are encoded by colors: red and green are used to represent positive and negative values, respectively. Individual log_2_ values ranged from –2.47 to 3.26.

To further identify discriminating molecular pathways that might be predictive of thyroid pathology and cancer risk, additional emphasis was placed on molecular pathways affected by both PB and PTU but not NaI. Interestingly, large differences were observed in transcripts from ribosomal protein and Wnt signaling gene families. Although not observed after NaI exposure, multiple 40S ribosomal protein transcripts were significantly reduced by PTU and PB treatment, including S4 (Rps4), S6 (Rps6), and S8 (Rps8) proteins (Table 6). NaI-dependent changes in ribosomal transcripts were observed in the large 60S subunit that included L22 (Rpl22) and L18 (Rpl18a) ribosomal proteins. Wnt target gene transcripts cyclin D1 (Ccnd1), c-jun (Jun), insulin-like growth factor binding protein (Igfbp2), VEGF receptor (Kdr), and protein kinase C (PKC) ζ(Prkcz) were up-regulated 50–300% in thyroids from PB- and PTU-treated animals, whereas no treatment-related changes in these transcripts were observed after NaI exposure (Table 6; Figure 3). PTU also altered the expression of other Wnt target genes, including multiple cyclin D3 (Ccnd3) transcripts and urokinase plasminogen activator receptor (Plau) mRNA (Table 6, Figure 3). Decreased expression of the adenomatous polyposis coli (Apc) gene and/or its homologs was also observed after exposure to PB and PTU. Finally, a modest increase (40%) in frizzled protein (Fzd1) mRNA was observed on a single gene target from the NaI treatment group. Multiple protein kinase transcripts were affected by PB and/or PTU exposure, including the up-regulation of Prkcz (PB and PTU) and PKCα(Prkca), and down-regulation of protein kinase A [Prkaa; GenBank accession no. X57986 (http://www.ncbi.nlm.nih.gov); PTU only]. In contrast, NaI did not significantly alter the transcript levels of any protein kinase (Table 6; Figure 3).

## Discussion

Many organic iodides alter rat thyroid homeostasis that may potentially lead to thyroid tumors after chronic administration (Capen 1997; Hurley et al. 1998). Although chemically induced changes in thyroid hormone metabolism and thyroid hormone release have been associated with increased thyroid tumor incidence in rodents, no such correlation with cancer risk has been associated with excess iodide (Capen 1997; Markou et al. 2001). To better characterize thyroid toxicity associated with increased cancer risk in the rat, genomic, clinical, and pathological end points were examined in response to excess NaI (non-carcinogen) and the rodent thyroid carcinogens PB and PTU in a modified 2-week endocrine battery (O’Connor et al. 2002).

Similar to previous reports, PB and PTU caused hormonal alterations (reduced serum T_3_, rT_3_, and T_4_ levels and increased TSH) and induced thyroid follicular cell hypertrophy in male rats after a 2-week exposure period (De Sandro et al. 1991). PB also increased relative liver weights (percent of body weight) and hepatic UDPGT activity, suggesting the involvement of altered thyroid hormone metabolism in PB-induced thyroid toxicity (Barter and Klassen 1992). In contrast, PTU reduced hepatic 5′-DI activity, increased relative thyroid weights (percent body weight), and induced thyroid follicular cell hyperplasia in male rats, suggesting an alternative mechanism of toxicity, including peripheral conversion of T_4_ to T_3_ (Oppenheimer et al. 1972). These effects elicited by PB and PTU in this short-term study, specifically, increases in TSH levels resulting in microscopic alterations of the thyroid gland, are suggestive of a potential thyroid tumor response after longer-term exposures in male rats (Capen 1997).

No biologically significant effects were observed after NaI exposure using the same treatment regimen. NaI caused no changes in thyroid hormone levels or thyroid histopathology. UDPGT activity was statistically significantly reduced in rats dosed with 100 mg/kg/day NaI, although liver weights were significantly increased in male rats receiving 10 and 100 mg/kg/day NaI. The significance of these effects on hepatic UDPGT activity is unclear but suggests an alternative effect on liver metabolism despite the concomitant increase in liver weights. These dose-dependent effects demonstrate for the first time that excess iodide can elicit a biological/toxicological response on the liver. The importance of these observed liver effects are also supported by the fact that iodide doses used in this study (1–10 mg/kg/day) are obtainable in high-dose toxicology studies with iodinated compounds.

We found multiple gene transcripts that were significantly altered by linear regression, regularized t-test, and principle component analysis models in response to NaI treatment. The transcripts most sensitive to iodide exposure included the thyroid NIS and GST-pi. NIS mRNA was significantly reduced to 40% of control levels at NaI doses as low as 1 mg/kg/day. A similar reduction in NIS mRNA has been reported previously after 6 days of exposure to 0.05% NaI in the drinking water of rats (Eng et al. 1999). In that study a concomitant reduction in protein level was also demonstrated suggestive of reduced iodide transport into the thyroid. In our study, GST-pi mRNA was significantly increased (150% of control) in the thyroid of rats exposed to the lowest dose of NaI tested (0.1 mg/kg/day). The biologic significance of increased GST-pi (Gstp2) transcripts in the rat thyroid is uncertain but may be predictive of increased oxidative stress. Other antioxidant gene transcripts were induced in the rat thyroid after NaI exposure, including Gpx1 and Gpx2 (1.4- and 2.4-fold, respectively), Sod2 (1.6 fold), Txnrd1 (1.5-fold), heme oxygenase (Hmox1, 1.9-fold), and NAD(P)H dehydrogenase quinone (Nqo1, 2.2-fold; Table 5). Iodide increases oxidative stress in cultured thyroid cells as evidenced by increased intra-cellular reactive oxygen species and lipid peroxidation (Vitale et al. 2000). It has been suggested that I_2_, the molecular form of ionic iodide, is highly reactive with protein, lipids, and nucleic acids and that generation of iodocompounds may disrupt cellular membrane functions, increase reactive oxygen species, and cause programmed cell death in thyroid cells. Dose-dependent increases in the antioxidant enzyme transcripts Gpx2 and Gstp2 observed in the rat thyroid are consistent with a compensatory response to the generation of lipid peroxides as reported by Vitale et al. (2000) using iodide exposures in vitro. The observed alterations in multiple transcripts involved in apoptosis in our studies are also consistent with this proposed mechanism of iodide-induced thyroid toxicity.

NaI exposure also influenced the mRNA expression of a wide range of rhodopsin-like G-protein–coupled receptors including adrenergic receptors. NaI increased β-adrenergic receptor (Adrb2) gene expression in a dose-dependent manner, whereas PB and PTU increased α_1_-adrenergic receptor (Adra1d) gene expression. In previous in vitro studies, thyrotropin (TSH) increased α1-adrenergic receptor mRNA in rat thyroid FRTL-5 cells as a compensatory mechanism to down-regulate TSH-induced cAMP levels (Corda and Kohn 1985). cAMP is considered the primary second messenger in thyroid follicular cell growth and maintenance (Medina and Santisteban 2000; Richards 2001). Together with the observed reduction in TSH receptor mRNA observed in the thyroids of rats exposed to PB and PTU, these gene expression changes are suggestive of an adaptive response to high levels of circulating TSH seen in rats treated with PB and PTU but not NaI. Further investigation is needed to understand the biological significance, if any, of β-adrenergic receptor mRNA induction by NaI.

To further identify gene expression changes that may be predictive of cancer risk, additional emphasis was placed on signaling pathways influenced by PB and PTU (carcinogens) but not NaI (noncarcinogen). Interestingly, significant changes were observed in gene expression patterns associated with Wnt signaling in PB and PTU exposed animals. Up-regulation of Ccnd1, Ccnd3, and Jun mRNA (all transcriptional targets of Wnt signaling) are suggestive of increased risk to human cancer (Behrens 2000; Ishigaki et al. 2002). Cyclin D1 protein is overexpressed in human thyroid cancers that have causal links to environmental radiation exposures at nuclear test sites (Meirmanov et al. 2003). Cyclin D1 and cyclin D3 both facilitate entry into the cell cycle, and increased transcription of these genes is associated with increased cell proliferation and hyperplasia (Baldassarre et al. 2003; Ishigaki et al. 2002). Thyroid hyperplasia was only observed in PTU animals and not PB animals (hypertrophy was observed in both groups), suggesting that increased expression of these genes precedes visible histopathological changes. Finally, the proto-oncogene Jun, which is also associated with multiple types of human cancer, mediates its oncogenic activity by stimulating AP-1–mediated transcription and cell proliferation (Shaulian and Karin 2001). Up-regulation of Jun has been associated with thyroid cancer, but its importance in mediating Wnt signaling in the thyroid has yet to be characterized (Battista et al. 1998).

Increased β-catenin protein is a common marker for Wnt signaling in multiple types of cancer, including thyroid cancer (Behrens 2000). β-Catenin complexes with the adenomatous polyposis coli (Apc) gene and axin to regulate its phosphorylation by glycogen synthase kinase (Gsk3b) and subsequent proteosomal degradation (Henderson and Fagotto 2002). Wnt activation inhibits the phosphorylation of β-catenin, resulting in its nuclear accumulation, binding to the lymphoid enhancer factor-1/T-cell–specific transcription factor (LEF-1/TCF), and transcription of multiple target genes as previously discussed (Kikuchi 2000). Interestingly, significantly lower gene expression levels of Apc and/or two other tumor suppressor proteins with homologous sequence mRNAs were observed in the thyroid glands of animals treated with PB and PTU but not NaI. In addition, mRNA expression of Prkcz, an inhibitor of Gsk3b kinase activity, was up-regulated in response to PB and PTU animals but not NaI (Oriente et al. 2001). Inhibition of Gsk3b results in decreased phosphorylation of β-catenin and causes its nuclear accumulation. Lastly, a DNA damage protein (Gadd45a) and a MAP kinase (Mapk14), two other proteins recently associated with Wnt activation, were also differentially expressed in PB- and PTU-treated animals but not in NaI-treated animals (Hildesheim et al. 2004).

In further support of our findings, recent investigations have also demonstrated silencing of Wnt signaling in mammalian cells after exposures to thyroid hormone (T_3_; Miller et al. 2001; Natsume et al. 2003). In these studies T_3_ inhibited β-catenin accumulation and transcriptional activity in rat pituitary and human kidney cells, respectively. Although T_3_ promotes cellular growth in many cellular systems, it is suggested that T_3_ involvement in normal organ development and cellular differentiation also implies antitumor activity of this critical hormone. In our studies, reductions in thyroid hormone were only observed in PB-and PTU-treated animals with the most significant changes after PTU exposure. This correlates well with the magnitude and number of Wnt signaling target genes changed in the microarray analyses of each treatment group.

Finally, significant changes in ribosomal protein mRNA were also observed in PB- and PTU-exposed animals. Alterations in ribosomal protein expression have been associated with increased cell proliferation and hyperplasia in multiple tissue types (Chou and Blenis 1995; Hamadeh et al. 2002). The ribosomal protein Rps6, regulated by p70 ribosomal S6 kinase (pp70s6k), is important in transcription and translation events precluding entry into the cell cycle (Cass and Meinkoth 1998). The mRNA of this protein was down-regulated in PB- and PTU-treated animals, correlating with other cAMP signaling responses (protein kinase mRNA regulation) observed in this study.

**Figure 4:**
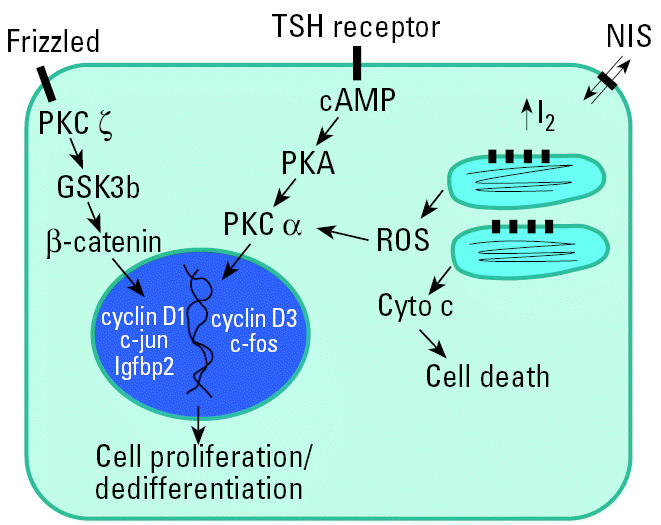
Increased circulating TSH activates protein kinase A (PKA) via the TSH receptor and the second-messenger cAMP in thyroid follicular cells. Cyto c, cytochrome *c*. We propose that decreased circulating thyroid hormone (T_3_), either by increasing T_4_ glucoronidation (PB) or decreasing peripheral conversion of T_4_ (PTU), modulates PKCζ activity and increases the transcription of Wnt target genes. Reactive oxygen species (ROS) generated by intracellular I_2_ after NaI or organic iodide metabolism may also increase protein kinase activity or cell death dependent on the magnitude of the oxidative stress and/or other extracellular signals.

In summary, we present new findings with regard to thyroid gene expression in the rat after subchronic exposure to NaI, PB, and PTU. Although no significant changes in biochemical and pathological measurements of thyroid function were observed in the rat after NaI exposure, significant changes in thyroid gene expression were observed even at the lowest concentration of NaI tested (0.1 mg/kg/day). Many of these genes, namely, multiple antioxidant enzymes have not been characterized previously in the rat thyroid and may prove useful as biomarkers of iodide exposure. We also demonstrate gene expression changes associated with thyroid histopathology and hormone status after PB and PTU exposures. Expression patterns of Wnt signaling genes correlated with circulating thyroid hormone levels, thyroid histopathology, and the carcinogenic potential of PB and PTU in the rat. The potential interactions between these mechanisms of thyroid toxicity are summarized in Figure 4. We suggest that organic iodides may elicit changes in circulating hormone levels as well as cause increases in oxidative stress in the rat thyroid. Although excess iodide may not cause follicular cell transformation via activation of Wnt signaling, it may enhance cytotoxicity in the rat thyroid exacerbated by thyroid pathology caused by fluctuations in thyroid hormone levels. These findings, in addition to further elucidating signaling pathways that correlate with rat thyroid pathology, may provide useful biomarkers for the prediction of thyroid tumorigenesis after chronic exposure to xenobiotics in the rat.

## Figures and Tables

**Table 1:** Thyroid hormone parameters.

Treatment (mg/kg/day)	T_3_ (ng/mL)	T_4_ (ng/mL)	TSH (ng/dL)	rT_3_ (ng/mL)
Water	73.6 ± 12.5	4.23 ± 0.83	12.2 ± 6.1	0.111 ± 0.015
NaI (0.1)	75.6 ± 12.4	4.37 ± 0.92	10.7 ± 3.6	0.118 ± 0.018
NaI (1)	69.0 ± 10.5	4.40 ± 1.01	11.5 ± 4.2	0.114 ± 0.022
NaI (10)	68.9 ± 10.4	4.38 ± 1.29	15.1 ± 7.8	0.106 ± 0.023
NaI (100)	73.3 ± 14.4	4.58 ± 1.14	13.1 ± 5.0	0.106 ± 0.020
PB (100)	56.5 ± 10.4*	2.52 ± 0.84*	22.4 ± 10.2*	0.080 ± 0.024*
PTU (10)	14.5 ± 7.3*	0.04 ± 0.10*	51.9 ± 12.4*	0.049 ± 0.018*

Values represent mean ± SD of 16 or more measurements from individual animals in each group.

*Statistical significance from control as determined by Jonckheere-Terpstra trend test, p < 0.05.

**Table 2:** Thyroid gland pathology.

Treatment (mg/kg/day)	Thyroid (g)	Colloid	Hypertrophy	Hyperplasia
Water	0.006 ± 0.001	–	–	—
NaI (100)	0.007 ± 0.002	–	–	—
PB (100)	0.007 ± 0.001	+	+	—
PTU (10)	0.018 ± 0.003*	++	++	++

Abbreviations: –, no lesions observed; +, lesions observed; ++, severe lesions observed. Values represent mean ± SD of 9–11 measurements from individual animals in each group.

*Statistical significance from control as determined by least significant difference and Dunnett’s test, p < 0.05.

**Table 3:** Principal component analysis.

Principal component	Eigenvalue	Proportion	Cumulative
1	2.53220382	0.4220	0.4220
2	1.36004986	0.2267	0.6487
3	0.69452426	0.1158	0.7645
4	0.51077061	0.0851	0.8496
5	0.49305017	0.0822	0.9318
6	0.40940129	0.0682	1.0000

**Table 4:** Principal component values based on treatment group.

Variable	Pcomp1	Pcomp2	Pcomp3	Pcomp4	Pcomp5	Pcomp6
Lnsigrat3	0.491944	–0.079240	0.067459	0.816707	–0.153903	–0.237625
Lnsigrat5	0.502641	–0.050801	0.241487	–0.396262	0.480074	–0.546773
Lnsigrat7	0.494279	0.023657	0.208137	–0.391877	–0.721315	0.194792
Lnsigrat9	0.498959	–0.063955	–0.394606	–0.007669	0.406380	0.652712
Lnsigrat11	0.109047	0.688894	–0.627580	–0.063206	–0.133869	–0.312667
Lnsigrat13	0.013435	0.715482	0.586721	0.135445	0.206111	0.287812

Abbreviations: Lnsigrat#, natural logarithm of the transcription signal ratio for treatment number; Pcomp#, principal component number.

**Table 5:** Treatment-related effects on rhodopsin-like G-protein–coupled receptor and oxidative stress–related gene expression.

Gene group/name^a^	Gene symbol^a^	GenBank accession no.^a^	NaI	PB	PTU
Rhodopsin-like GPCRs					
Alpha-1D adrenergic receptor	Adra1d	M60654	—c	1.6	3.0
Alpha-1B adrenergic receptor	Adra1b	M60655	2.0	2.1	3.4
Alpha-1C adrenergic receptor	Adra1c	U13368	–2.1	—c	—c
Alpha-2A adrenergic receptor	Adra2a	U79031	1.9	—	—
Beta-2 adrenergic receptor	Adrb2	J03024	2.9	—	—
Beta-3 adrenergic receptor	Adrb3	S56481	–2.2	—	–2.1
Serotonin receptor 6	Htr6	S62043	–1.6	—	—
Serotonin receptor 4	Htr4	U20907	—	—	4.1
Serotonin receptor 1F	Htr1f	L05596	—	7.7	—
Serotonin receptor 7	Htr7	L22558	—	—	2.5
Dopaminergic receptor D-3	Drd3	A17753	—	3.4	—
Opioid receptor mu-1	Oprm1	D16349	—	–2.7	—
Cholinergic receptor muscarinic 3	Chrm3	M16407	—	—	–1.7
Cholinergic receptor muscarinic 5	Chrm5	M22926	–1.9	—	—
Neuropeptide Y5 receptor	Npy5r	U66274	2.1	—	—
Panceatic polypeptide receptor	Ppyr1	U42388	–2.1	—	—
Interleukin 8 receptor beta	Il8rb	U70988	—	—	–2.0
Chemokine receptor 4	Cxcr4	U90610	—	—	1.9
Chemokine receptor 1	Cx3cr1	U04808	—	—	2.1
A3 adenosine receptor	—b	X93219	–2.8	—	—
Angiotensin II receptor	—	M90065	—	—	–1.8
Angiotensin II receptor (AT1B)	—	X64052	—	–3.7	—
Thyroid-stimulating hormone receptor	Tshr	M34842	—	–1.6	–1.7
Chemokine orphan receptor 1	Cmkor1	AJ010828	1.5	—	—
Endothelin receptor type B	Ednrb	X57764	—	—	1.6
Prostaglandin E receptor 4	Ptger4	D28860	—	—	3.0
Platelet-activating factor receptor	Ptafr	U04740	—	—	2.6
Bradykinin receptor B1	Bdkrb1	AJ132230	–1.4	—	—
GABAB receptor 1	Gabbr1	AB016161	–1.6	—	—
Olfactory receptor pseudogene	Olr1469	AF091570	–1.3	—	—
Olfactory receptor 1696	Olr1696	AF034896	–1.7	—	—
Olfactory receptor 1699	Olr1699	AF034899	–2.9	—	—
Olfactory receptor 226	Olr226	M64386	—	—	–1.9
Olfactory receptor 1361	Olr1361	M64377	–2.0	—	—
Olfactory receptor 1370	Olr1370	AF091577	–2.2	—	—
Olfactory receptor 1493	Olr1493	AF091572	–2.4	—	—
Olfactory receptor 1346	Olr1346	AF091578	–4.2	—	—
Olfactory receptor 1687	Olr1687	AF091563	–5.3	–3.6	—
Oxidative stress–related genes					
NAD(P)H dehydrogenase quinone	Nqo1	J02679	2.2	1.5	2.0
Heme oxygenase 1	Hmox1	J02722	1.9	2.0	—
Glutamate-cysteine ligase	Gclc	J05181	1.5	1.5	—
Thioredoxin reductase 1	Txnrd1	U63923	1.5	—	—
Glutathione peroxidase 1	Gpx1	X07365	1.4	—	1.4
Glutathione peroxidase 2	Gpx2	AA800587	2.4	—	—
Glutathione reductase	Gpr	U73174	—	1.6	1.6
GST-pi	Gstp2	X02904	2.5	2.0	2.6
Superoxide dismutase 3	Sod3	X68041	–1.4	—	1.7
Superoxide dismutase 2	Sod2	Y00497	1.6	—	—
Superoxide dismutase 1	Sod1	M25157	–2.0	—	—
Metallothionein-1a	Mt1a	M11794	–1.7	–1.9	–2.1
Peroxiredoxin 1	Prdx1	AI010083	1.2	—	—
Nitric oxide synthase 2	Nos2	U16359	2.2	—	—
Cytochrome b-245 alpha	Cyba	U18729	1.7	—	1.6
Monoamine oxidase A	—	S45812	—	—	–1.4

Values represent statistically significant (p < 0.01 by regularized t-test) mean fold change from control (n = 3) for 100 mg/kg/day NaI and PB and 10 mg/kg/day PTU.

a From GenBank (http://www.ncbi.nlm.nih.gov).

b —, Gene symbol unknown.

c —, Not statistically significant.

**Table 6:** Treatment-related effects on Wnt signaling and ribosomal protein gene expression.

Gene group/names^a^	Gene symbol^a^	GenBank accession no.^a^	NaI	PB	PTU
Wnt signaling genes					
APC fragment 2 of 6	—b	L19304^c^	—d	–1.6	—d
APC fragment 4 of 6	—	L19306	—	–2.2	–2.6
APC protein	Apc	D38629	—	—	–2.0
c-jun oncogene	Jun	X17163	—	2.9	2.3
Cyclin D1	Ccnd1	D14014	—	1.4	2.6
Cyclin D1 partial	Ccnd1	X75207^c^	—	1.3	2.3
Cyclin D3	Ccnd3	D16309^c^	—	1.4	1.7
Cyclin D3 partial	Ccnd3	U49935^c^	—	1.6	1.7
Frizzled homolog 1	Fzd1	L02529	1.5	—	—
DNA damage inducible transcript	Gadd45a	L32591	—	–1.7	–2.0
Insulin-like factor binding protein	Igfbp2	M91595	—	1.7	5.2
Low-density lipoprotein receptor	Ldlr	X13722	—	—	2.1
p38 MAP kinase	Mapk14	U91847	—	1.6	—
p38 MAP kinase 2	Mapk14	U73142	—	—	1.4
Protein kinase C, alpha	Prkca	X07286	—	—	3.7
Protein kinase C, zeta	Prkcz	M18332	—	1.6	1.4
Plasminogen activator, urokinase	Plau	X63434	—	—	–1.8
VEGF receptor 2	Kdr	U93306	—	1.9	2.8
Ribosomal protein genes					
Ribosomal protein S5	Rps5	X58465	—	—	–1.4
Ribosomal protein S4	Rps4	X14210	—	–1.4	–1.4
Ribosomal protein S6	Rps6	M29358	—	–1.5	–1.2
Ribosomal protein S9	Rps9	X66370	—	—	–1.3
Ribosomal protein S8	Rps8	X06423	—	–1.3	–1.3
Ribosomal protein S7	Rps7	X53377	—	—	–1.3
Ribosomal protein S3	Rps3	X51536	—	—	–1.3
40 kDa ribosomal protein	Lamr1	D25224	—	—	–1.3
v-fos transformation effector	Rps3a	M84716	—	–1.6	–1.2
Ribosomal protein L3	Rpl3	X62166	—	—	–1.4
Ribosomal protein L5	Rpl5	M17419	—	–1.5	—
Ribosomal protein L9	Rpl9	X51706	—	—	–1.4
Ribosomal protein L10	Rpl10	X93352	—	—	–1.4
Ribosomal protein L11	Rpl11	X62146	—	—	–1.4
Ribosomal protein L12	Rpl12	X53504	—	—	–1.4
Ribosomal protein L14	Rpl14	X94242	—	—	–1.3
Ribosomal protein L17	Rpl17	X58389	—	—	–1.4
Ribosomal protein S25	Rps25	X62482	—	—	–1.4
Ribosomal protein L7	Rpl7a	X15013	—	—	–1.3
Ribosomal protein L18	Rpl18a	X14181	–1.4	—	—
Ribosomal protein L21	Rpl21	M27905	—	–1.4	–1.4
Ribosomal protein L22	Rpl22	X60212	–2.1	—	–1.8
Ribosomal protein L23	Rpl23	X65228	—	—	–1.5
Ribosomal protein L26	Rpl26	X14671	—	—	–1.4
Ribosomal protein L28	Rpl28	X52619	—	—	–1.3
Ribosomal protein L37	Rpl37	X66369	—	—	—

Values represent statistically significant (p < 0.01 by regularized t-test) mean fold change from control (n = 3) for 100 mg/kg/day NaI and PB and 10 mg/kg/day PTU.

a From GenBank (http://www.ncbi.nlm.nih.gov).

b —, Gene symbol not known.

c p ≤ 0.03 by regularized t-test.

d —, Not statistically significant.
